# Diagnosis and Prevention of Periprosthetic Joint Infections by Staphylococcus aureus after Hip Fracture: A Systematic Review of the Literature

**DOI:** 10.1055/s-0043-1776019

**Published:** 2024-03-21

**Authors:** Bianca Gabriella de Oliveira, Victor Hugo Ruis da Costa, Igor Rodrigues Gama, Murilo Halberstadt Beskow, Elisson Rafael Silva dos Santos

**Affiliations:** 1 Departamento da Liga Acadêmica de Ortopedia e Traumatologia da UNIFACS, Universidade Salvador (UNIFACS), Salvador, BA, Brasil; 2 Serviço de Ortopedia e Traumatologia, Hospital Universitário de Canoas, Canoas, RS, Brasil

**Keywords:** arthroplasty, replacement, hip, hip prosthesis, *staphylococcal* infections

## Abstract

Hip arthroplasties are surgical procedures widely performed all over the world, seeking to return functionality, relieve pain, and improve the quality of life of patients affected by osteoarthritis, femoral neck fractures, osteonecrosis of the femoral head, among other etiologies. Periprosthetic joint infections are one of the most feared complications due to the high associated morbidity and mortality, with a high number of pathogens that may be associated with its etiology. The aim of the present study was to analyze aspects correlated with the occurrence of infection, diagnosis and prevention of periprosthetic joint infections in the hip associated with *Staphylococcus aureus* after corrective surgery for hip fractures. This is a systematic review of the literature carried out in the databases indexed in the Medical Literature Analysis and Retrieval System Online (MEDLINE) carried out in accordance with the precepts established by the Preferred Reporting Items for Systematic Reviews and Meta-Analyses (PRISMA) methodology. Twenty studies that addressed the diagnosis and prevention of periprosthetic joint infections after hip fractures were selected for analysis. It is observed that there is no consensus in the literature on preventive measures for the occurrence of such infectious processes. Among the risk factors for the occurrence and severity of infections by *S. aureus* after hip arthroplasties, obesity, longer surgical time, older age, immunosuppression, recent use of antibiotics, and multicomorbidities were mentioned. The use of biomarkers for early diagnosis, as well as screening, decolonization, and antibiotic prophylaxis processes are among the preventive procedures proposed in the literature.

## Introduction

Hip arthroplasty has extensive clinical application for pain relief and functional improvement, especially in patients with osteoarthritis, osteonecrosis of the femoral head, and femoral neck fracture. Although it is a procedure already enshrined in the surgical literature, it is observed that the clinical results are associated with multiple factors such as the surgical procedure, perioperative management, prosthetic design, existing comorbidities, as well as other individual factors. 1 2

Among the risks associated with the placement of a prosthesis in the hip joint, the literature points to a higher risk of venous thromboembolism (VTE) and periprosthetic joint infection (PJI) 1 as the most frequent complications and also with the greatest potential for increased morbidity and mortality and cost in public health. 3

The occurrence of an infectious process after hip arthroplasties is characterized as one of the most feared complications, both due to the possible need for surgical reapproach, as well as the risk of worsening the infectious condition, need to remove the prosthesis, and the impact of the entire process on the health and functionality of the patient. Periprosthetic joint infections still represent a challenge in medical practice, from early diagnosis, identification of causative pathogens, and proper management. 4 5

Although there are several clinical guidelines associated with the prevention of peri- and postoperative complications, it is observed that such normative divergence causes high rates of clinical variation in the management of patients undergoing hip arthroplasties, also resulting in different clinicians.

In view of the lack of consensus on preventive management of periprosthetic joint infections and the multiplicity of pathogens that may be involved in the infectious process, the present study aimed to analyze aspects correlated with the occurrence of infection, diagnosis, and prevention of PJIs in the hip associated with *Staphylococcus aureus* after corrective surgery for hip fractures.

## Materials and methods

The present study is characterized as a systematic literature review, structured according to the guidelines of Preferred Reporting Items for Systematic Reviews and Meta-Analyses (PRISMA) 6 and subsequently structured a PRISMA checklist for analysis of results. A four-phase diagram was used to choose the articles, prioritizing clarity and transparency in the execution of the systematic review and selection of studies.

The data search took place on June 5, 2022, in the databases linked to the Medical Literature Analysis and Retrieval System Online (MEDLINE), using the Setting, Perspective, Intervention, Comparison, and Evaluation (SPICE) 7 strategy to identify relevant studies:

- Setting: Hospitalized patients
- Perspective: Individuals with hip fracture evaluated for PJI
- Intervention: Total or partial hip replacement surgery
- Comparison: Occurrence of *S. aureus* infection, diagnosis, screening, and prevention.
- Evaluation: Rate or occurrence of periprosthetic joint infection by *S. aureus* , biomarkers, screening, and effectiveness of decontamination.

Health sciences descriptors (DECS) / MESH TERMS were used in combination, according to the following structures: *Arthroplasty* OR *Hip Prosthesis* AND *Staphylococcal Infections* OR *Infecção por Staphylococcus aureus* .

### Inclusion and exclusion criteria

Studies that met the following criteria were included: (1) human studies, age group > 18 years old; (2) patients undergoing hip arthroplasty after fracture; (3) studies addressing patients with PJI by *S. aureus* ; (4) studies published between 2017 and 2022; and (5) original studies.

Studies with the following criteria were excluded: (1) experimental studies with animal models; (2) non-original studies – literature review; (3) opinion studies; (4) studies that addressed conduct after established infection, that is, that did not discuss prevention and diagnosis of the infectious condition; (5) studies published > 5 years ago; and (6) studies that did not meet the other aforementioned inclusion criteria.

The search and selection of studies was conducted by two reviewers who independently performed the analysis of the studies. Initially, from the use of the mentioned DECS, together with Boolean operators, studies published in the last 5 years (2017 to 2022) were selected, followed by the analysis of titles and abstracts. At this stage, studies with animal models, opinion articles, as well as literature reviews were excluded.

Once this step was completed, the complete texts of the articles were retrieved for analysis of the other inclusion and exclusion criteria. Duplicate citations and studies not corresponding to the proposed review parameters were also excluded. Possible disagreements were resolved through discussion with a third reviewer, with inclusion decided after consensus with the two main reviewers.

Seeking to prioritize methodological quality, studies classified as “Good” after the National Institutes of Health (NIH) quality assessment were included, and studies with more than nine checked items were considered suitable for inclusion.

The extraction of epidemiological and demographic data was performed using a Microsoft Excel 365 (Microsoft Corporation, Redmond, WA, USA) spreadsheet, including parameters such as number of patients, surgical approach, risk factors described, and infection prevention strategies.

## Results

In the screening process using the proposed DECS and respective Boolean Operators, 245 studies were initially retrieved. Subsequently, when excluding studies published > 5 years ago, 65 articles remained for title and abstract analysis. Seven studies were excluded because they were literature reviews. After reading the abstracts, another 28 studies were excluded, totaling 30 studies for full text reading.

Ten studies were excluded because they addressed surgical procedures associated with prosthetic replacement after an infectious condition and did not discuss aspects associated with the diagnosis and prevention of periprosthetic infection by *S. aureus.*

After reading the texts in full, 20 studies were selected for discussion. It is noteworthy that although some studies addressed both knee and hip periprosthetic joint infections, it was decided to discuss the data related to infections that affect the hip joint, as this is the central theme of the present construct.

**Fig. 1:**
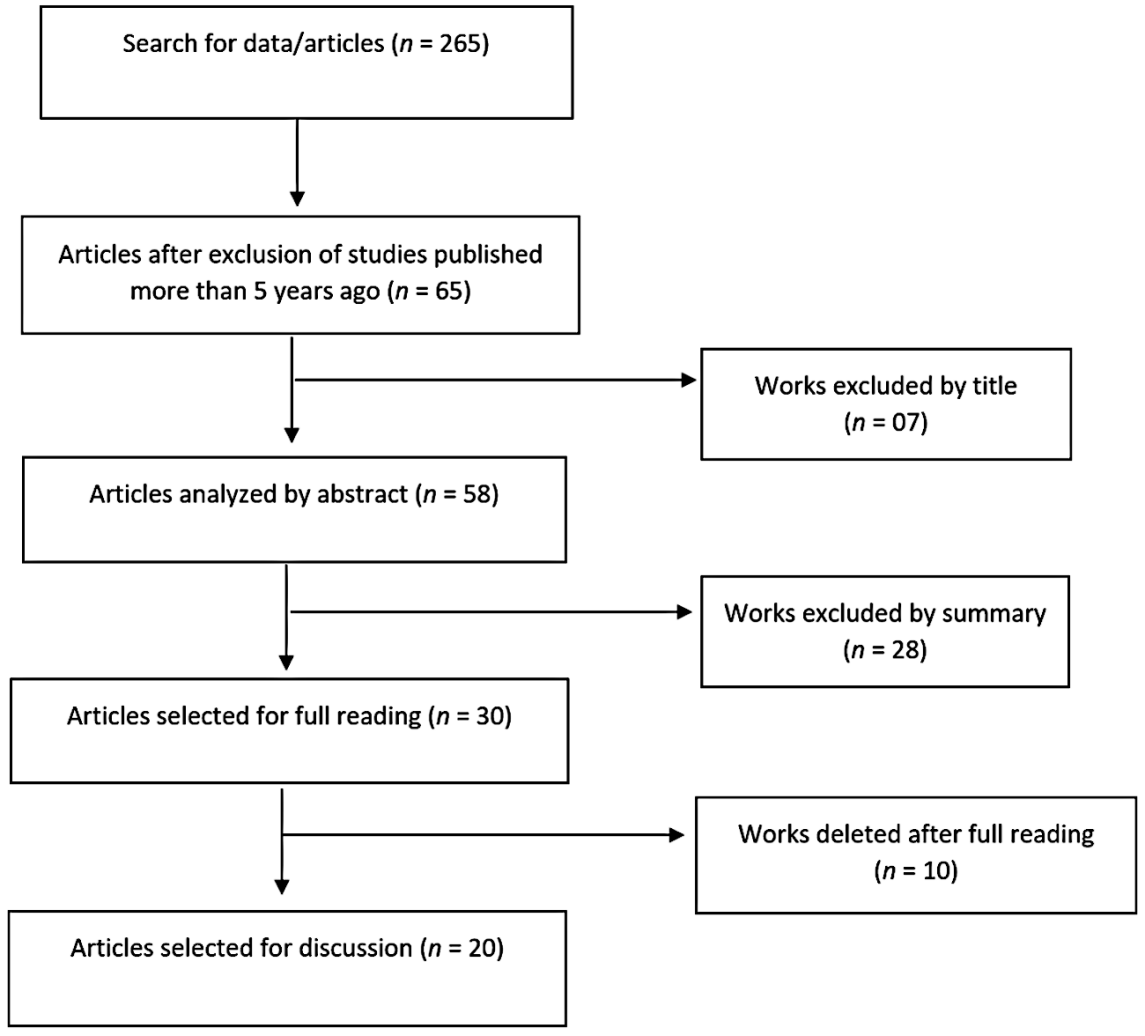
Flowchart of screening and selection of studies according to the PRISMA methodology. **Source:** Own elaboration (2021).

Fig. 1 shows the flowchart of the study selection process, as proposed by the PRISMA methodology adopted in the present study.

Periprosthetic joint infections represent a major challenge in the management of patients undergoing orthopedic surgeries. There are several risk factors and pathogens involved in the etiology of infections, with relevant literature discussion on diagnostic methods, biomarkers, screening, and viability of decolonization of these patients.

Table 1 briefly describes the studies selected for discussion considering author, year of publication, study scope, prevalence of reported colonization, and relevant aspects.

**Table 1:** Brief description of the studies selected for discussion

Author	Year	Sample	Coverage	Prevalence of Colonization	Relevant aspects
Ascione et al. 35	2017	122 PJI	Single center - Italy	*S. aureus* - 36% CoNS - 34%	- Presence of comorbidities increased the risk of PJI
Banke et al. 14	2020	25 PJI	Single center - Germany	MMSA - 100% (study bias)	- Antimicrobial peptides as PJI biomarkers
Barbero et al. 32	2017	307 patients	Single center - Spain	*S. aureus* - 28.3%	- Decolonization with intranasal mupirocin and chlorhexidine baths effective for PJI reduction.
Bauer et al. 15	2020	340 patients	Single center - France	*S. aureus* - 21%	- Gold standard intraoperative tissue culture for infectious definition - Measurement of serum anti-staphylococcal antibodies increased positive predictive value of synovial fluid culture for diagnosis of PJI
Çimen et al. 34	2020	354 surgeons	Single center - Turkey	Variable not addressed	- Time of antibiotic prophylaxis longer than proposed by guidelines - Blood glucose measurement performed by 94% of the sample -Decolonization for MRSA performed by 33.3%
Guo et al. 20	2020	96 PJI	Single center - China	*S. epidermidis* – 38.10%	- Risk factors for PJI: obesity, longer surgical time, and immunosuppression.
Hariharan et al. 13	2019	22 patients	Single center - India	*S. aureus* - 18%	- ESR > 30mm/h had a sensitivity of 75% and specificity of 88% in predicting PJI. - CRP > 10mg/L had a sensitivity of 75% and a specificity of 69%.
Hartman et al. 12	2022	158 PJI	Single center - EUA	MSSA - 19.6%	- CRP was a marker of reinfection in patients with PJI
Morgenstern et al. 10	2018	142 patients	Single center - Germany	CoNS - 14.33% S. aureus - 19%	- PJI in 54% sample - Aseptic failure 46%
Papalini et al. 8	2022	63 PJI	Single center - Italy	*S. aureus* - 20.3% CONS - 20.3% Streptococcus spp. - 7.5%	- Approves EBJIS criteria - Suggests combination of techniques for diagnosing PJI.
Pietrazak et al. 21	2020	119 patients	Single center – South Africa	MSSA - 31.9% MRSA - 0%	- Combined intranasal mupirocin ointment and chlorhexidine body wash for decolonization.
Rohrer et al. 26	2021	1318 patients	Single center - Switzerland	*S. aureus* - 34%	- PJI index of 0.3%
Rosteius et al. 19	2018	477 patients	Single center - Germany	MSSA - 28.2% MRSE - 13.2% MRSA - 6.6% CoNS -16.4%	- Risk factors for PJI: multicomorbidities, advanced age and obesity.
Scholten et al. 27	2020	10486 patients	Single center - Netherland	*S. aureus* - 26.7%	- Nasal screening and eradication protocol did not reduce global PJI but reduced early *S. aureus* -induced PJI.
Schweitzer et al. 17	2021	146 patients	Single center - Chile	MRSA - 5%	- Larger MRSA colonization in the inguinal region - Recent use of antibiotics as an important risk factor
Stambough et al. 33	2017	4.186 patients	Single center - USA	MRSA - 0.09%	- Universal decolonization reduced PJI and surgical site infections
Tani et al. 9	2018	114 patients	Single center - Greece	Variable not addressed	- Culture sensitivity of the sonication fluid of 77.04%, - Sensitivity of conventional tissue cultures of 55.73%
Tonotsuka et al. 25	2021	1654 patients	Single center - Japan	*S. aureus* – 26.9% MRSA – 1.8%	- Combined intranasal mupirocin ointment for decontamination - Female sex as a risk factor for greater colonization by S. aureus
Tsai et al. 18	2019	159 PJI	Single center - Taiwan	*S. aureus* - 27% MRSA - 21% CoNS -14%	- Empirical antibiotic therapy with wide coverage until PJI culture.
Villa et al. 16	2021	361 PJI	Multicenter - North America / South America and Europe	*S. aureus* - 24.8% *S. epidermidis* - 21.7% Polymicrobial - 10.5%	- 62.3% resistant to at least 1 antibiotic.

Abbreviations: CoNS, Staphylococcus coagulase negative; MRSA, methicillin-resistant Staphylococcus aureus; MRSE, methicillin-resistant Staphylococcus epidermidis; MSSA, methicillin-sensitive Staphylococcus aureus; PJI, Patients undergoing hip arthroplasty with periprosthetic joint infection; S., Staphylococcus.

## Discussion

### Biomarkers and Diagnosis of PJI

The first challenge imposed on the medical team regarding PJI is precisely their diagnosis. The literature points out that there is a lack of sensitive tests for the diagnosis and there is no consensus on the methodology used for causal definition and establishment of the definitive diagnosis. 8 9 10

The European Bone and Joint Infection Society (EBJIS) proposes, as of 2021, a three-level diagnostic approach considering an unlikely infection, probable infection, and confirmed infection. To be defined as a confirmed infection, one must observe a sinus tract with evidence of communication to the joint or visualization of the prosthesis or leukocyte count > 3,000 or Polymorphonuclear (PMN) > 80% or positive alpha-defensin immunoassay, or positive methods of microbiological study by aspiration or sonication, or even histological study showing microorganisms or increased neutrophils. 11

On the other hand, cases of probable infection are defined by 2 positive findings, including radiological signs, aspects of compromised healing, bacteremia, fever, periprosthetic purulence, increased C-reactive protein (CRP), leukocyte count > 1,500, PMN > 65%, positive cultures, in addition to histological study and suggestive nuclear images. Infection is unlikely when all findings are negative. 11

In a study carried out in Italy, 133 patients with PJI, 63 with hip infection, were evaluated according to the criteria proposed by the EBJIS for diagnosing PJI. The authors emphasized that the diagnostic tests need to be used together under the risk of erroneously discarding a PJI condition. It was observed that according to the EBJIS criteria, 101 diagnoses would be confirmed and 25 would be described as probably infected, which demonstrates the applicability of the criteria when used together. Regarding the probable etiology of the PJI, a prevalence of 20.3% of *S. aureus* , 20.3% of coagulase-negative Staphylococcus (CoNS), and 7.5% of Streptococcus spp. was observed. 8

When evaluating 158 patients treated for PJI, it was observed that 19.6% ( *n* = 31) suffered reinfection within 2 years. There was a statistically significant association between Methicillin-susceptible Staphylococcus aureus (MSSA) infection and reinfection. Considering possible infection markers that help in early diagnosis, the authors highlighted an increase in the mean serum CRP level of 12.65 g/dL, while patients without reinfection had a mean serum CRP of 5.0 g/dL. 12 In an Indian study, the sensitivity and specificity of using erythrocyte sedimentation rate (ESR) and CRP cutoff points in the diagnosis of infection were 57 and 94%, respectively, corroborating these results. 13

When compared with the CRP of the synovial fluid, the culture of the same material showed less clinical applicability due to the delay in obtaining the result. The sensitivity of synovial fluid culture and CRP was 52 and 60%, respectively, showing concordant results in 82% of the sample. C-reactive protein was superior for detection of low virulence bacteria such as Cutibacterium spp. and coagulase-negative staphylococci. 10

Another PJI biomarker described in the literature was antimicrobial peptides (AMPs). According to the researchers, significant intra-articular levels of human cathelicidin LL-37 and ß-defensin-3 (HBD-3) have high diagnostic accuracy in the synovial fluid of patients with PJI. Synoviocytes isolated as a source of cellular AMP showed comparable results with a significant increase in LL-37/HBD-3 up to 3 × in PJI. However, it is noteworthy that the study sample ( *n* = 25) was extremely small, representing an important gap to support the results found, as well as the need to analyze costs associated with the procedure and clinical applicability in other centers. 14

Preoperative culture of synovial fluid combined with serum anti-staphylococcal antibody dosage was also chosen for better accuracy in the diagnosis and etiological definition of PJI. 15 Still correlated to the diagnostic accuracy by different types of obtaining culture medium, it was observed in a study carried out in Greece that the sensitivity of the sonication fluid culture was 77.04%, and the sensitivity of conventional tissue cultures was 55.73%, with respective specificities of 98.11 and 94.34%. In view of these results, the authors point out that the sonication method represents a reliable test for diagnosing PJI, with greater sensitivity and specificity than conventional periprosthetic tissue cultures. 9

### Epidemiology

Of the total of 20 selected studies, it was observed that 5 studies addressed general epidemiological issues on the subject, discussing microbiological aspects, prevalence, and incidence of periprosthetic hip joint infections. Of these, only one study was multicenter involving health institutions in North/South America and Europe. The other studies addressed contexts in Chile, South Africa, Taiwan, and Germany.

In a multicenter study carried out by Villa et al., 16 the authors considered 654 periprosthetic infections, 361 in the hip and 293 in the knee. It was observed that the most frequently identified microorganisms were *S. aureus* (24.8%) and *Staphylococcus epidermidis* (21.7%). As for the degree of resistance to at least 1 antibiotic, the authors identified a general index of 58%, and in hip infections this index was higher, of 62.3%. The overall incidence of polymicrobial infections was 9.3% in knees and hips pooled and 10.5% in hips alone. 16

Analyzing the occurrence of colonization by resistant microorganisms, the study carried out in Chile with 146 patients undergoing hip arthroplasty indicated that at least 5% of these were colonized by methicillin-resistant *S. aureus* (MRSA); according to the authors, cutaneous and nasal colonization by MRSA is associated with a higher incidence of infection after surgeries to place a hip prosthesis. They also highlighted that individuals with recent use of antibiotics are more likely to have MRSA colonization. 17

In a study carried out in a single center in Taiwan, the main pathogens associated with PJI in the hip were identified. Of the total of 159 registered cases, 27% were caused by *S.* *aureus* , 14% by CoNS, 21% by MRSA, and 4.1% by fungi and mycobacteria. Given the high prevalence of colonization and diversity of pathogens, the authors suggest empirical antibiotic therapy with broader coverage in the period between the identification of the infectious condition and the PJI culture result. 18

A similar study carried out in Germany with 477 patients with PJI also showed *S. aureus* as the main pathogen involved (28.2%), followed by methicillin-resistant *S. epidermidis* (MRSE; 13.2%), MRSA (6.6%), CoNS (16.4%), and streptococcus (9.1%). In the study, the researchers consider that the existence of multicomorbidities, advanced age, and obesity are conditions of greater risk for the appearance of PJI. 19

Other risk factors for PJI described in the literature were: prolonged surgical time, previous use of immunosuppressants, history of previous surgery on the incisions, preoperative hypoproteinemia, and superficial infection. 20

A study with 96 patients who suffered PJI indicated that the main etiological agents were Gram-positive pathogens. *Staphylococcus epidermides* was the most common after hip arthroplasties (38.10%) while *S. aureus* was the main infectious agent found in PJI of the knee joint (40.74%). 20 Other analyzed studies also indicated that highly resistant pathogens such as MSSA, 17 21 MRSE, 16 18 19 MRSA, CoNS, Enterococcus, and Streptococcus are responsible for most periprosthetic infections, with MRSA and MRSE being the most common infections when analyzing pathogens resistant to antibiotic therapy. 18 19 22 23 24

### Nasal screening and decolonization

The literature describes several strategies for nasal screening and decolonization aimed at reducing the occurrence of PJI after hip arthroplasty. 25 26 27 Universal decolonization proposes decolonization for all patients without a screening process, while targeted decolonization provides for a screening process to identify patients eligible for decolonization. Although screening has been referred to as the most effective strategy from an economic point of view, there is great difficulty in identifying individuals who would be eligible for screening, that is, those at higher risk of PJI.

In a study carried out in Japan with 1,654 patients undergoing total hip arthroplasty, the researchers did not identify independent predictive factors for contamination by MRSA, and only female gender was identified as an independent risk factor for *S. aureus* . Thus, it would not be possible to identify useful predictive parameters for nasal screening, thus requiring universal decolonization in patients undergoing the surgical procedure. 25

Ten studies addressed preoperative decolonization procedures as a way to prevent the occurrence of PJI. In all cases, topical mupirocin (ointment) was used in the nostrils in the decolonization protocol, applied 2 to 3 times a day for 5 days, associated with daily baths with 2 to 4% chlorhexidine. 21 25 26 27 28 29 30 31 32 33 Of these, only three emphasized the effectiveness of decolonization to reduce PJI, and in one of the studies there was a reduction only in early PJI caused by *S. aureus* , not reducing global PJI. 27 In the other two studies, there was a reduction in overall PJI 32 33 and one of the studies also showed a reduction in surgical site infections in patients with preoperative decolonization. 33

As reported by Barbero et al., 32 *S. aureus* is the main agent causing infections in joint prostheses. In a control study involving 307 patients with hip fractures who underwent elective arthroplasties, colonization by *S. aureus* was observed in 28.3% of the sample. We opted for a *S. aureus* detection-decolonization protocol with intranasal mupirocin and chlorhexidine baths, with a considerable reduction in the incidence of PJI in the decolonized group. 32

Contrary to what was described in the previous study, research carried out in Switzerland with 1,318 patients undergoing hip prosthetic surgery failed to show significant differences between the groups colonized with *S. aureus* and noncolonized with regard to the occurrence of PJI (0.3%). In view of these findings, the authors declared that over 2 years, since no PJI had occurred in neither group, there would be no possibility of a definitive conclusion on the effectiveness of preoperative decolonization, although the incidence of PJI is lower than that of other studies found in the literature. 26

Three studies considered the costs associated with performing universal decolonization. In a North American study, it was observed that the use of the universal decolonization protocol, that is, without prior screening, was able to reduce the incidence of surgical site infections (5 versus 15 cases; 0.2 versus 0.8%; *p* = 0.013) and also of PJI associated with *S. aureus* . In view of the results, it was considered that the cost-effectiveness would be valid, representing economic gains and lower morbidity and mortality associated with infections after arthroplasties. 33 In both cases, useful predictive parameters for the implementation of the screening strategy were not identified, considering that universal decolonization would be effective and appropriate in cases of patients undergoing hip arthroplasties. 25 29 33

Among the selected studies, antibiotic prophylaxis was also addressed, discussing aspects related to the type of drug to be used, as well as variables considered for initiation and discontinuation. In Turkey, it was observed that most surgeons (56.8%) use antibiotic prophylaxis for a longer period than proposed in the guidelines (> 24 hours), glycemic assessment before surgery was performed by 94% of the sample, and decolonization for MRSA was performed by 33.3% of study participants. 34 Timely administration of antibiotics and absence of comorbidities were associated with a favorable outcome in both prevention and control of PJI .35

## References

[1] Huang X T Liu D G Jia B Xu Y X Comparisons between direct anterior approach and lateral approach for primary total hip arthroplasty in postoperative orthopaedic complications: a systematic review and meta-analysis Orthop Surg 2021 13 06 1707 1720 34351056 10.1111/os.13101 PMC8523754

[2] Cochrane N H Kim B I Wu M O'Donnell J A Seidelman J L Jiranek W A Cutibacterium Positive Cultures in Total Hip Arthroplasty: Contaminant or Pathogen? J Arthroplasty 2022 37(7S):S642 S646 35660199 10.1016/j.arth.2022.01.015

[3] Badge H M Churches T Naylor J M Xuan W Armstrong E Gray L Non-compliance with clinical guidelines increases the risk of complications after primary total hip and knee joint replacement surgery PLoS One 2021 16 11 e0260146 34793555 10.1371/journal.pone.0260146 PMC8601457

[4] Gundtoft P H Pedersen A B Schønheyder H C Møller J K Overgaard S One-year incidence of prosthetic joint infection in total hip arthroplasty: a cohort study with linkage of the Danish Hip Arthroplasty Register and Danish Microbiology Databases Osteoarthritis Cartilage 2017 25 05 685 693 27986623 10.1016/j.joca.2016.12.010

[5] Prattingerová J Sarvikivi E Huotari K Ollgren J Lyytikäinen O Surgical site infections following hip and knee arthroplastic surgery: Trends and risk factors of Staphylococcus aureus infections Infect Control Hosp Epidemiol 2019 40 02 211 213 30522540 10.1017/ice.2018.312

[6] Liberati A Altman D G Tetzlaff J Mulrow C Gøtzsche P C Ioannidis J PA The PRISMA statement for reporting systematic reviews and meta-analyses of studies that evaluate health care interventions: explanation and elaboration J Clin Epidemiol 2009 62 10 e1 e34 19631507 10.1016/j.jclinepi.2009.06.006

[7] Booth A Searching for qualitative research for inclusion in systematic reviews: a structured methodological review Syst Rev 2016 5 74 27145932 10.1186/s13643-016-0249-x PMC4855695

[8] Papalini C Pucci G Cenci G Mencacci A Francisci D Caraffa A Prosthetic joint infection diagnosis applying the three-level European Bone and Joint Infection Society (EBJIS) approach Eur J Clin Microbiol Infect Dis 2022 41 05 771 778 35318542 10.1007/s10096-022-04410-x PMC9033695

[9] Tani S Lepetsos P Stylianakis A Vlamis J Birbas K Kaklamanos I Superiority of the sonication method against conventional periprosthetic tissue cultures for diagnosis of prosthetic joint infections Eur J Orthop Surg Traumatol 2018 28 01 51 57 28714050 10.1007/s00590-017-2012-y

[10] Morgenstern C Cabric S Perka C Trampuz A Renz N Synovial fluid multiplex PCR is superior to culture for detection of low-virulent pathogens causing periprosthetic joint infection Diagn Microbiol Infect Dis 2018 90 02 115 119 29191466 10.1016/j.diagmicrobio.2017.10.016

[11] McNally M Sousa R Wouthuyzen-Bakker M Chen A F Soriano A Vogely H C The EBJIS definition of periprosthetic joint infection Bone Joint J 2021 103-B 01 18 25 10.1302/0301-620X.103B1.BJJ-2020-1381.R1 PMC7954183 33380199

[12] Hartman C W Daubach E C Richard B T Lyden E R Haider H Kildow B J Predictors of reinfection in prosthetic joint infections following two-stage reimplantation J Arthroplasty 2022 37(7S):S674 S677 35283230 10.1016/j.arth.2022.03.017

[13] Hariharan T D Chandy V J George J Mathew A J Premnath J Pragasam A K Microbiological profile and outcomes of two-stage revision hip arthroplasty Indian J Med Microbiol 2019 37 01 67 71 31424013 10.4103/ijmm.IJMM_19_25

[14] Banke I J Stade N Prodinger P M Tübel J Hapfelmeier A von Esenhart-Rothe R Antimicrobial peptides in human synovial membrane as (low-grade) periprosthetic joint infection biomarkers Eur J Med Res 2020 25 01 33 32799924 10.1186/s40001-020-00434-1 PMC7429885

[15] Bauer T Marmor S Ghout I Salomon E El Sayed F Heym B Measurement of serum anti-staphylococcal antibodies increases positive predictive value of preoperative aspiration for hip prosthetic joint infection Clin Orthop Relat Res 2020 478 12 2786 2797 32667753 10.1097/CORR.0000000000001392 PMC7899396

[16] Villa J M Pannu T S Theeb I Buttaro M A Oñativia J I Carbo L International Organism Profile of Periprosthetic Total Hip and Knee Infections J Arthroplasty 2021 36 01 274 278 32828620 10.1016/j.arth.2020.07.020

[17] Schweitzer D Klaber I García P López F Lira M J Botello E Methicillin-resistant *Staphylococcus aureus* colonization in patients undergoing primary total hip arthroplasty J Med Microbiol 2020 69 04 600 604 32427561 10.1099/jmm.0.001155

[18] Tsai Y Chang C H Lin Y C Lee S H Hsieh P H Chang Y Different microbiological profiles between hip and knee prosthetic joint infections J Orthop Surg (Hong Kong) 2019 27 02 2.309499019847768E15 10.1177/2309499019847768 31117922

[19] Rosteius T Jansen O Fehmer T Baecker H Citak M Schildhauer T A Evaluating the microbial pattern of periprosthetic joint infections of the hip and knee J Med Microbiol 2018 67 11 1608 1613 30207518 10.1099/jmm.0.000835

[20] Guo H Xu C Chen J Risk factors for periprosthetic joint infection after primary artificial hip and knee joint replacements J Infect Dev Ctries 2020 14 06 565 571 32683346 10.3855/jidc.11013

[21] Pietrzak J RT Maharaj Z Mokete L Prevalence of Staphylococcus aureus colonization in patients for total joint arthroplasty in South Africa J Orthop Surg Res 2020 15 01 123 32238194 10.1186/s13018-020-01635-4 PMC7110725

[22] Zawadzki N Wang Y Shao H Liu E Song C Schoonmaker M Readmission due to infection following total hip and total knee procedures: A retrospective study Medicine (Baltimore) 2017 96 38 e7961 28930833 10.1097/MD.0000000000007961 PMC5617700

[23] Cunningham D J Kavolus J J II Bolognesi M P Wellman S S Seyler T M Specific infectious organisms associated with poor outcomes in treatment for hip periprosthetic infection J Arthroplasty 2017 32 06 1984 1.99E8 28222919 10.1016/j.arth.2017.01.027 PMC5440199

[24] Canadian Nosocomial Infection Surveillance Program Roth V R Mitchell R Vachon J Alexandre S Amaratunga K Smith S Periprosthetic infection following primary hip and knee arthroplasty: the impact of limiting the postoperative surveillance period Infect Control Hosp Epidemiol 2017 38 02 147 153 27834161 10.1017/ice.2016.256

[25] Tonotsuka H Sugiyama H Amagami A Yonemoto K Sato R Saito M What is the most cost-effective strategy for nasal screening and Staphylococcus aureus decolonization in patients undergoing total hip arthroplasty? BMC Musculoskelet Disord 2021 22 01 129 33522920 10.1186/s12891-021-04008-y PMC7849129

[26] Rohrer F Wendt M Noetzli H Risch L Bodmer T Cottagnoud P Preoperative decolonization and periprosthetic joint infections-A randomized controlled trial with 2-year follow-up J Orthop Res 2021 39 02 333 338 33258495 10.1002/jor.24916

[27] Scholten R Hannink G Willemsen K Mascini E M Somford M P Schreus B W Preoperative *Staphylococcus aureus* screening and eradication Bone Joint J 2020 102-B 10 1341 1348 32993339 10.1302/0301-620X.102B10.BJJ-2020-0038.R1

[28] Tandon T Tadros B J Akehurst H Avasthi A Hill R Rao M Risk of Surgical Site Infection in Elective Hip and Knee Replacements After Confirmed Eradication of MRSA in Chronic Carriers J Arthroplasty 2017 32 12 3711 3717 28739308 10.1016/j.arth.2017.06.036

[29] Rennert-May E Conly J Smith S Puloski S Henderson E Au F A cost-effectiveness analysis of mupirocin and chlorhexidine gluconate for *Staphylococcus aureus* decolonization prior to hip and knee arthroplasty in Alberta, Canada compared to standard of care Antimicrob Resist Infect Control 2019 8 113 31338160 10.1186/s13756-019-0568-5 PMC6625116

[30] Jeans E Holleyman R Tate D Reed M Malviya A Methicillin sensitive staphylococcus aureus screening and decolonisation in elective hip and knee arthroplasty J Infect 2018 77 05 405 409 29932962 10.1016/j.jinf.2018.05.012

[31] Kerbel Y E Sunkerneni A R Kirchner G J Prodromo J P Moretti V M The cost- effectiveness of preoperative staphylococcus aureus screening and decolonization in total joint arthroplasty J Arthroplasty 2018 33(7S):S191 S195 29510950 10.1016/j.arth.2018.01.032

[32] Barbero J M Romanyk J Vallés A Plasencia M A Montero E López J [Decolonization for Staphylococcus aureus carriers in arthroplasty surgery after hip fracture] Rev Esp Quimioter 2017 30 04 264 268 28585795

[33] Stambough J B Nam D Warren D K Keeney J A Clohisy J C Barrack R L Decreased Hospital Costs and Surgical Site Infection Incidence With a Universal Decolonization Protocol in Primary Total Joint Arthroplasty J Arthroplasty 2017 32 03 728 7340 27823845 10.1016/j.arth.2016.09.041

[34] Çimen O Azboy N Çatal B Azboy İ Assessment of periprosthetic joint infection prevention methods amongst Turkish orthopedic surgeons in total joint replacement: A survey Jt Dis Relat Surg 2020 31 02 230 237 32584719 10.5606/ehc.2020.71425 PMC7489147

[35] Ascione T Pagliano P Balato G Mariconda M Rotondo R Esposito S Oral therapy, microbiological findings, and comorbidity influence the outcome of prosthetic joint infections undergoing 2-stage exchange J Arthroplasty 2017 32 07 2239 2243 28372916 10.1016/j.arth.2017.02.057

