# Supplementary material for: Diagnosis and Prevention of Periprosthetic Joint Infections by Staphylococcus aureus after Hip Fracture: A Systematic Review of the Literature
Source: Rev Bras Ortop (Sao Paulo). 2024 Mar 21;59(1):e21–8. doi: 10.1055/s-0043-1776019 (PMC10957261; doi:10.1055/s-0043-1776019)
Supplement: Related texts

# Research article: Diagnóstico e prevenção de infecções articulares periprotéticas por *Staphylococcus aureus* após fratura de quadril: Revisão sistemática de literatura

**Keywords:** artroplastia de quadril, prótese de quadril, infecções estafilocócicas

## Abstract

As artroplastias de quadril são procedimentos cirúrgicos largamente realizados em todo o mundo buscando retorno da funcionalidade, alívio da dor e melhora da qualidade de vida dos pacientes acometidos por quadros de osteoartrite, fraturas de colo de fêmur e osteonecrose da cabeça femoral, dentre outras etiologias. As infecções articulares periprotéticas são uma das complicações mais temidas pela elevada morbimortalidade associada, com elevado número de patógenos que podem estar associados à sua etiologia. O objetivo do presente estudo foi analisar aspectos correlacionados à ocorrência da infecção, diagnóstico e prevenção de infecções articulares periprotéticas no quadril associadas a *Staphylococcus aureus* após cirurgia corretiva de fraturas de quadril. Trata-se de uma revisão sistemática de literatura realizada nas bases de dados indexadas na Medical Literature Analysis and Retrieval System Online (MEDLINE, na sigla em inglês) realizada de acordo com os preceitos estabelecidos pela metodologia Preferred Reporting Items for Systematic Reviews and Meta-Analyses (PRISMA, na sigla em inglês). Foram selecionados para análise 20 estudos que abordavam o diagnóstico e prevenção de infecções articulares periprotéticas após fraturas de quadril. Observa-se que não há consenso na literatura sobre medidas preventivas para ocorrência de tais processo infecciosos. Dentre os fatores de risco para ocorrência e gravidade das infecções por *S. aureus* após artroplastias de quadril foram citados obesidade, maior tempo cirúrgico, maior idade, quadros de imunossupressão, uso recente de antibióticos e multicomorbidades. O uso de biomarcadores para diagnóstico precoce, bem como processos de triagem, descolonização e antibioticoprofilaxia estão entre os procedimentos preventivos propostos na literatura.

## Introdução

A artroplastia do quadril possui extensa aplicação clínica no alívio da dor e melhora funcional, sobretudo em pacientes com quadros de osteoartrite, osteonecrose da cabeça femoral e fratura de colo de fêmur. Embora seja um procedimento já consagrado na literatura cirúrgica, observa-se que os resultados clínicos estão associados a múltiplos fatores, como o procedimento cirúrgico, manejo perioperatório, desenho protético, comorbidades existentes, bem como demais fatores individuais. 1 2

Dentre os riscos associados à colocação de prótese na articulação do quadril, a literatura aponta o maior risco de tromboembolismo venoso (TEV) e de infecção articular periprotética (IAP) 1 como as complicações de maior ocorrência e também com maior potencial de aumento de morbimortalidade e custo em saúde pública. 3

A ocorrência de processo infeccioso após artroplastias de quadril caracteriza-se como uma das complicações mais temidas tanto pela possível necessidade de reabordagem cirúrgica como também pelo risco de piora do quadro infeccioso, necessidade de retirada da prótese e impacto de todo processo para a saúde e funcionalidade do paciente. As IAPs representam ainda um desafio na prática médica desde o seu diagnóstico precoce, identificação dos patógenos causadores e manejo adequado. 4 5

Embora existam diversas diretrizes clínicas associadas à prevenção de complicações peri- e pós-operatórias, observa-se que tal divergência normativa ocasiona altas taxas de variações no manejo de pacientes submetidos a artroplastias de quadril, resultando também em diferentes desfechos clínicos.

Frente à ausência de consenso sobre o manejo preventivo de infecções articulares periprotéticas e à multiplicidade de patógenos que podem estar envolvidos no processo infecioso, o presente estudo teve como objetivo analisar aspectos correlacionados à ocorrência da infecção, diagnóstico e prevenção de infecções articulares periprotéticas no quadril associadas a *Staphylococcus aureus* após cirurgia corretiva de fraturas de quadril.

## Materiais e métodos

O estudo caracteriza-se como uma revisão sistemática de literatura, estruturada conforme as orientações de Preferred Reporting Items for Systematic Reviews and Meta-Analyses (PRISMA, na sigla em inglês), 6 sendo posteriormente estruturada uma lista de verificação PRISMA para análise dos resultados. Utilizou-se ainda um diagrama de quadro fases, para escolha dos artigos, priorizando clareza e transparência na execução da revisão sistemática e seleção dos estudos.

A busca de dados ocorreu em 05 de junho de 2022, nos bancos de dados vinculados à Medical Literature Analysis and Retrieval System Online (MEDLINE, na sigla em inglês), sendo utilizada a estratégia Setting, Perspective, Intervention, Comparison, and Evaluation (SPICE, na sigla em inglês) 7 para identificação dos estudos relevantes:

- Setting (Cenário): pacientes hospitalizados
- Perspective (Perspectiva): indivíduos com fratura de quadril avaliados quanto à IAP
- Intervention (Intervenção): Cirurgia de colocação de prótese total ou parcial em quadril
- Comparison (Comparação): ocorrência de infecção por *S. aureus* , diagnóstico, triagem e prevenção.
- Evaluation (Avaliação): Taxa ou ocorrência de infecção articular periprotética por *S. aureus* , biomarcadores, triagem e efetividade da descontaminação.

Os descritores em ciências da saúde (DECS)/MESH TERMS foram utilizados de forma combinada, conforme as seguintes estruturas: *Artroplastia de Quadril* ( *Arthroplasty* ) OR *Prótese de Quadril* ( *Hip Prosthesis* ) AND *Infecções Estafilocócicas* ( *Staphylococcal Infections* ) OR *Infecção por Staphylococcus aureus* .

## Critérios de inclusão e exclusão

Foram incluídos estudos que atendiam aos seguintes critérios: (1) estudos com humanos, grupo etário > 18 anos (2) pacientes submetidos a artroplastia do quadril após fratura (3) estudos abordando pacientes com infecção articular periprotética por *S. aureus* (4) estudos publicados entre 2017-2022 (5) estudos originais.

Foram excluídos estudos com os seguintes critérios: (1) estudos experimentais com modelos animais (2) estudos não originais – revisão de literatura (3) estudos de opinião (4) estudos que abordavam condutas após infecção estabelecida, ou seja, que não discutiam prevenção e diagnóstico do quadro infeccioso (5) estudos publicados há mais de 5 anos (6) estudos que não atendiam aos demais critérios de inclusão supracitados.

A busca e seleção dos estudos se deu por dois revisores que realizaram independentemente a análise dos estudos. Inicialmente, a partir da utilização dos DECS mencionados, juntamente com operadores Booleanos, foram selecionados estudos publicados nos últimos 5 anos (2017-2022), seguido da análise de títulos e resumos. Nesta etapa, foram excluídos estudos com modelos animais, artigos de opinião, bem como revisões de literatura.

Concluída tal etapa, procedeu-se à recuperação dos textos completos dos artigos para análise dos demais critérios de inclusão e exclusão. Foram excluídos ainda citações duplicadas e estudos não correspondentes aos parâmetros revisionais propostos. Possíveis discordâncias foram solucionadas com discussão com um terceiro revisor, sendo a inclusão decidida após consenso com os dois revisores principais.

Buscando primar por qualidade metodológica, foram incluídos estudos classificados como “Bons” após avaliação de qualidade do National Institutes of Health (NIH, na sigla em inglês), sendo que estudos com mais de nove itens assinalados eram considerados aptos para serem incluídos.

A extração dos dados epidemiológicos e demográficos foi realizada com auxílio de planilha do Microsoft Excel 365 (Microsoft Corporation, Redmond, WA, EUA), incluindo parâmetros como número de pacientes, abordagem cirúrgica, fatores de risco descritos e estratégias de prevenção de infecção.

## Resultados

No processo de triagem, utilizando-se os DECS propostos e os respectivos operadores Booleanos, foram recuperados inicialmente 245 estudos. Posteriormente, ao excluir estudos publicados há mais de 5 anos, restaram 65 artigos para análise do título e resumo. Sete estudos foram excluídos por se tratar de revisões de literatura. Após a leitura dos resumos, outros 28 estudos foram excluídos, totalizando 30 estudos para leitura do texto na íntegra.

Dez estudos foram excluídos por abordarem procedimentos cirúrgicos associados à troca protética após quadro infeccioso e não discutirem aspectos associados ao diagnóstico e à prevenção da infecção periprotética por *S. aureus* .

Após a leitura dos textos na íntegra, foram selecionados então 20 estudos para discussão. Destaca-se que embora alguns estudos abordassem tanto infecções articulares periprotéticas do joelho como do quadril, optou-se por discutir os dados relativos às infecções que acometem a articulação do quadril, por ser esta a temática central do presente constructo.

Na Fig. 1 , está representado o fluxograma do processo de seleção dos estudos, conforme proposto pela metodologia PRISMA adotada no presente estudo.

**Fig.1:** Fluxograma de triagem e seleção de estudos conforme a metodologia PRISMA. **Fonte:** Autoria própria (2021).

As IAPs representam um grande desafio no manejo de pacientes submetidos a cirurgias ortopédicas. Diversos são os fatores de risco e patógenos envolvidos na etiologia das infecções, tendo ainda relevante discussão da literatura sobre métodos diagnósticos, biomarcadores, triagem e viabilidade da descolonização destes pacientes.

Na Tabela 1 , estão descritos sucintamente os estudos selecionados para discussão considerando autor, ano de publicação, abrangência do estudo, prevalência de colonização referida e aspectos relevantes.

**Tabela 1:** Descrição sucinta dos estudos selecionados para discussão

Autor	Ano	Amostra	Abrangência	Prevalência de Colonização	Aspectos relevantes
Ascione et al. 35	2017	122 IAPs	Unicêntrico - Itália	*S. aureus* – 36% ECN – 34%	Presença de comorbidades aumentou risco de IAP
Banke et al. 14	2020	25 IAPs	Unicêntrico - Alemanha	MMSA - 100% (Viés do estudo)	Peptídeos antimicrobianos como biomarcadores de IAP
Barbero et al. 32	2017	307 pacientes	Unicêntrico - Espanha	*S. aureus* - 28,3%	Descolonização com mupirocina intranasal e banhos de clorexidina efetiva para redução de IAP.
Bauer et al. 15	2020	340 pacientes	Unicêntrico - França	*S. aureus* - 21%	– Cultura de tecidos intraoperatória padrão-ouro para definição infecciosa – Dosagem de anticorpos antiestafilocócicos séricos – Aumento valor preditivo positivo da cultura do líquido sinovial para diagnóstico de IAP
Çimen et al. 34	2020	354 cirurgiões	Unicêntrico - Turquia	Variável não abordada	– Tempo de antibioticoprofilaxia superior ao proposto por diretrizes – Dosagem de glicemia realizada por 94% da amostra –Descolonização para MRSA realizada por 33,3%
Guo et al. 20	2020	96 IAP	Unicêntrico - China	*S. epidermidis* – 38,10%	– Fatores de risco para IAP: obesidade, maior tempo cirúrgico e imunossupressão.
Hariharan et al. 13	2019	22 pacientes	Unicêntrico - Índia	*S. aureus* – 18%	– VHS > 30mm/h teve uma sensibilidade de 75% e especificidade de 88% na previsão de IAP. –PCR > 10mg/L teve sensibilidade de 75% e especificidade de 69%.
Hartman et al. 12	2022	158 IAPs	Unicêntrico - EUA	MSSA – 19,6%	– PCR foi marcador de reinfecção em pacientes com IAP
Morgenstern et al. 10	2018	142 pacientes	Unicêntrico - Alemanha	ECN – 14,33% *S. aureus* – 19%	– IAP em 54% da amostra – Falha asséptica de 46%
Papalini et al. 8	2022	63 IAPs	Unicêntrico - Itália	S. aureus – 20,3% ECN – 20,3% Streptococcus spp. – 7,5%	– Aprova critérios da EBJIS – Sugere combinação de técnicas para diagnóstico de IAP.
Pietrazak et al. 21	2020	119 pacientes	Unicêntrico - África do Sul	MSSA – 31,9% MRSA – 0%	– Pomada de mupirocina intranasal combinada e lavagem corporal com clorexidina para descolonização.
Rohrer et al. 26	2021	1318 pacientes	Unicêntrico - Suiça	S. aureus – 34%	– Índice de IAP de 0,3%
Rosteius et al. 19	2018	477 pacientes	Unicêntrico - Alemanha	MSSA – 28,2% MRSE – 13,2% MRSA – 6,6% ECN -16,4%	– Fatores de risco para IAP: multicomorbidades, idade avançada e obesidade.
Scholten et al. 27	2020	10486 pacientes	Unicêntrico - Holanda	S. aureus – 26,7%	– Protocolo de rastreamento e erradicação nasal não reduziu IAP global, mas reduziu IAP precoce induzida por *S. aureus.*
Schweitzer et al. 17	2021	146 pacientes	Unicêntrico - Chile	MRSA – 5%	– Colonização de MRSA maior na região inguinal – Uso recente de antibióticos como fator de risco importante
Stambough et al. 33	2017	4.186 pacientes	Unicêntrico - EUA	MRSA – 0,09%	– Descolonização universal reduziu IAP e infecções do sítio cirúrgico
Tani et al. 9	2018	114 pacientes	Unicêntrico - Grécia	Variável não abordada	– Sensibilidade da cultura do fluido de sonicação de 77,04%, – Sensibilidade das culturas de tecidos convencionais de 55,73%
Tonotsuka et al. 25	2021	1654 pacientes	Unicêntrico - Japão	*S. aureus* – 26,9% MRSA – 1,8%	– Pomada de mupirocina intranasal combinada para descontaminação – Sexo feminino como fator de risco para maior colonização por *S. aureus*
Tsai et al. 18	2019	159 IAPs	Unicêntrico - Taiwan	*S. aureus* – 27% MRSA – 21% ECN –14%	–Antibioticoterapia empírica com ampla cobertura até cultura de IAP.
Villa et al. 16	2021	361 IAPs	Multicêntrico – América do Norte/América do Sul e Europa	*S. aureus* – 24,8% *S. epidermidis* – 21,7% Polimicrobianas – 10,5%	–62,3% resistentes a pelo menos 1 antibiótico.

Abreviações: ECN, Staphylococcus coagulase negativa; IAP, pacientes submetidos à artroplastia de quadril com infecção articular periprotética; MRSA, Staphylococcus aureus resistente à meticilina; MRSE, Staphylococcus epidermidis resistente à meticilina; MSSA, Staphylococcus aureus sensíveis à meticilina; S., Staphylococcus.

1 Utiliza-se no estudo a denominação “infecção articular periprotética” e sigla IAP como tradução livre do termo *Periprosthetic joint infection - PJI.*

## Discussão

## Biomarcadores e Diagnóstico de IAP

O primeiro desafio imposto à equipe médica no que se refere às IAPs é justamente o diagnóstico destas. A literatura pontua que faltam testes sensíveis para o diagnóstico e não há consenso na metodologia empregada para definição causal e estabelecimento do diagnóstico definitivo. 8 9 10

A European Bone and Joint Infection Society (EBJIS, na sigla em inglês) propõe, a partir de 2021, a abordagem diagnóstica de 3 níveis considerando um quadro de infecção improvável, infecção provável e infecção confirmada. Para ser definida como infecção confirmada, deve-se observar um trato sinusal com evidência de comunicação à articulação ou visualização da prótese ou contagem de leucócitos > 3.000 ou polimorfonucleares (PMN) > 80% ou imunoensaio positivo de alfa-defensina, ou métodos de estudo microbiológico por aspiração ou sonificação positivos, ou ainda estudo histológico apresentando microrganismos ou aumento de neutrófilos. 11

Já os quadros de infecção provável são definidos por dois achados positivos incluindo sinais radiológicos, aspectos de cicatrização comprometida, bacteremia, quadro febril, purulência periprotética, aumento da proteína C-reativa (PCR), contagem leucocitária > 1.500, PMN > 65%, culturas positivas, além de estudo histológico e imagens nucleares sugestivas. A infecção é improvável quando todos os achados são negativos. 11

Em um estudo realizado na Itália, 133 pacientes com IAP, sendo 63 com infecção do quadril, foram avaliados quanto aos critérios propostos pela EBJIS para diagnóstico de IAP. Os autores ressaltaram que os testes diagnósticos precisam ser utilizados em conjunto sob o risco de descartar erroneamente um quadro de IAP. Observou-se que, segundo os critérios da EBJIS, 101 diagnósticos seriam confirmados e 25 seriam descritos como provavelmente infectados, o que demonstra a aplicabilidade dos critérios desde que utilizados em conjunto. Quanto à etiologia provável das IAPs, observou-se prevalência de 20,3% de *S. aureus* , 20,3% de Estafilococos coagulase-negativos (ECN) e 7,5% de Streptococcus spp. 8

Quando avaliados 158 pacientes tratados para IAP, observou-se que 19,6% ( *n* = 31) sofreram reinfecção em um prazo de 2 anos. Houve associação estatisticamente significativa entre infecção por *staphylococcus aureus* suscetível à meticilina (MSSA) e reinfecção. Considerando possíveis marcadores de infecção que auxiliam no diagnóstico precoce, os autores destacaram aumento do nível médio de PCR sérica de 12,65 g/dL, enquanto pacientes sem reinfecção possuíam uma PCR sérica média de 5,0 g/dL. 12 Em um estudo indiano, a sensibilidade e a especificidade do uso de pontos de corte de velocidade de hemossedimentação (VHS) e PCR no diagnóstico de infecção foram de 57 e 94%, respectivamente, corroborando tais resultados. 13

Ao ser comparada com a PCR do líquido sinovial, a cultura do mesmo material se mostrou com menor aplicabilidade clínica pela demora na obtenção do resultado. A sensibilidade da cultura do líquido sinovial e PCR foi de 52 e 60%, respectivamente, mostrando resultados concordantes em 82% da amostra. A PCR foi superior para detecção de bactérias de baixa virulência, como Cutibacterium spp. e estafilococos coagulase-negativos. 10

Outro biomarcador de IAP descrito pela literatura foram os peptídeos antimicrobianos (AMPs, na sigla em inglês). Segundo os pesquisadores, níveis intra-articulares significativos de catelicidina humana LL-37 e ß-defensina-3 (HBD-3) apresentam alta acurácia diagnóstica no líquido sinovial de pacientes com IAP. Sinoviócitos isolados como fonte de AMP celular mostraram resultados comparáveis com um aumento significativo de LL-37/HBD-3 até 3 × em IAP. Contudo, destaca-se que a amostra do estudo ( *n* = 25) foi extremamente reduzida, representando uma importante lacuna para respaldar os resultados encontrados, bem como existe a necessidade de analisar custos associados ao procedimento e aplicabilidade clínica em demais centros. 14

Também foi elencado para melhor acurácia no diagnóstico e definição etiológica de IAP a cultura pré-operatória do líquido sinovial combinada com a dosagem de anticorpos antiestafilocócicos séricos. 15 Ainda correlacionado à acurácia diagnostica por diferentes tipos de obtenção de meio para cultura, observou-se em estudo realizado na Grécia que a sensibilidade da cultura do fluido de sonicação foi de 77,04%, e a sensibilidade das culturas de tecidos convencionais foi de 55,73%, com respectivas especificidades de 98,11 e 94,34%. Frente a tais resultados, os autores ressaltam que o método de sonicação representa um teste confiável para diagnóstico de IAP, com maior sensibilidade e especificidade do que culturas de tecidos periprotéticos convencionais. 9

## Epidemiologia

Do total de 20 estudos selecionados, observou-se que cinco estudos abordavam questões epidemiológicas gerais sobre a temática, discutindo aspectos microbiológicos, prevalência e incidência de infecções articulares periprotéticas de quadril. Destes, apenas um estudo era multicêntrico, envolvendo instituições de saúde da América do Norte/Sul e Europa. Os demais estudos abordavam contextos do Chile, África do Sul, Taiwan e Alemanha.

Em estudo multicêntrico realizado por Villa et al., 16 os autores consideraram 654 infecções periprotéticas, sendo 361 de quadril e 293 de joelho. Observou-se que os microrganismos mais frequentes identificados foram *S. aureus* (24,8%) e *Staphylococcus epidermidis* (21,7%). Quanto ao grau de resistência a pelo menos um antibiótico, os autores identificaram um índice geral de 58%, sendo que em infecções no quadril tal índice foi superior, sendo de 62,3%. A incidência geral de infecções polimicrobianas foi de 9,3% em joelhos e quadris agrupados e de 10,5% em análise exclusiva dos quadris. 16

Analisando a ocorrência de colonização de microrganismos resistentes, o estudo desenvolvido no Chile com 146 pacientes submetidos a artroplastia de quadril indicou que pelo menos 5% destes estavam colonizados por *S. aureus* resistente à meticilina (MRSA, na sigla em inglês); segundo os autores, a colonização cutânea e nasal por MRSA está associada à maior incidência de infecção após cirurgias para colocação de prótese no quadril. Os autores ressaltaram ainda que indivíduos com uso recente de antibióticos possuem maior probabilidade de colonização por MRSA. 17

Em estudo realizado em centro único de Taiwan, foram identificados os principais patógenos associados à IAP em quadril. Do total de 159 casos registrados, 27% foram causados por *S. aureus* , 14% por estafilococos coagulase-negativos (CoNS, na sigla em inglês), 21% por MRSA e 4,1% por fungos e micobactérias. Diante da elevada prevalência de colonização e da diversidade de patógenos, os autores sugerem antibioticoterapia empírica com cobertura mais ampla no período decorrido entre a identificação do quadro infeccioso e o resultado de cultura da IAP. 18

Estudo similar realizado na Alemanha com 477 pacientes com IAP também apresentou o *S. aureus* como principal patógeno envolvido (28,2%), seguido por *S. epidermidis* resistente à meticilina (MRSE, na sigla em inglês; 13,2%), MRSA (6,6%), CoNS (16,4%) e estreptococcus (9,1%). No estudo, os pesquisadores ponderam que a existência de multicomorbidades, idade avançada e obesidade são condições de maior risco para o surgimento de IAP. 19

Outros fatores de risco para IAP descritos na literatura foram: tempo cirúrgico prolongado, uso prévio de imunossupressores, história de cirurgia pregressa nas incisões, hipoproteinemia pré-operatória e infecção superficial. 20

Estudo com 96 pacientes que sofreram IAP indicou que os principais agentes etiológicos eram patógenos Gram-positivos. O *S. epidermides* foi o mais comum após artroplastias de quadril (38,10%) enquanto o *S. aureus* foi o principal agente infecioso encontrado em IAP da articulação do joelho (40,74%). 20 Outros estudos analisados também apontaram que patógenos de alta resistência como o MSSA, 17 21 MRSE, 16 18 19 MRSA, CoNS, Enterococcus e Streptococcus são responsáveis pela maior parte das infecções periprotéticas, sendo que MRSA e MRSE são comumente as infecções mais encontradas quando analisados patógenos resistentes à antibioticoterapia. 18 19 22 23 24

## Triagem nasal e descolonização

A literatura descreve diversas estratégias para triagem nasal e descolonização visando reduzir a ocorrência de IAP após artroplastia do quadril. 25 26 27 A descolonização universal propõe a descolonização para todos os pacientes sem processo de triagem, enquanto a descolonização alvo prevê um processo de triagem para identificação dos pacientes elegíveis para descolonização. Embora a realização de triagem tenha sido referida como a estratégia mais efetiva do ponto de vista econômico, observa-se grande dificuldade em identificar indivíduos que seriam elegíveis para triagem, ou seja, aqueles com maior risco de IAP.

Em estudo realizado no Japão com 1,654 pacientes submetidos à artroplastia total do quadril, os pesquisadores não identificaram fatores preditivos independentes para contaminação por MRSA, e apenas o sexo feminino foi identificado como fator de risco independente para *S. aureus* . Desta forma, não seria possível a identificação de parâmetros preditivos úteis para a triagem nasal, exigindo assim a descolonização universal nos pacientes submetidos ao procedimento cirúrgico. 25

Dez estudos abordaram procedimentos de descolonização pré-operatória como forma de prevenção da ocorrência de IAP. Em todos os casos, foram utilizados no protocolo de descolonização mupirocina tópica (pomada) nas narinas com aplicação de 2 a 3 vezes ao dia durante 5 dias, associado a banhos diários com clorexidina a 2–4%. 21 25 26 27 28 29 30 31 32 33 Destes, apenas três ressaltaram a efetividade da descolonização para redução de IAP, sendo que em um dos estudos houve redução apenas da IAP precoce causada por *S. aureus* , não reduzindo a IAP global. 27 Nos outros dois estudos, houve redução da IAP global 32 33 e ainda um dos estudos mostrou redução de infecções do sítio cirúrgico nos pacientes com descolonização pré-operatória. 33

Conforme relatado por Barbero et al. 32 o *S. aureus* é o principal agente causador de infecções em próteses articulares. Em estudo-controle envolvendo 307 pacientes com fratura de quadril submetidos a artroplastias eletivas, observou-se colonização por *S. aureus* em 28,3% da amostra. Optou-se por um protocolo de detecção-descolonização de *S. aureus* com mupirocina intranasal e banhos de clorexidina, havendo considerável redução da incidência de IAP no grupo descolonizado. 32

Contrariamente ao descrito pelo estudo anterior, uma pesquisa desenvolvida na Suíça com 1,318 pacientes submetidos a cirurgia protética de quadril não conseguiu evidenciar diferenças significativas entre os grupos colonizados com *S. aureus* e não colonizados no que se refere à ocorrência de IAP (0,3%). Diante de tais achados, os autores declararam que ao longo de 2 anos, por não ter ocorrido nenhuma IAP nos dois grupos, não haveria possibilidade de conclusão definitiva sobre a eficácia da descolonização pré-operatória, embora a incidência de IAP seja inferior aos demais estudos encontrados na literatura. 26

Três estudos consideraram os custos associados à realização de descolonização universal. Em um estudo Norte-Americano, observou-se que a utilização do protocolo de descolonização universal, ou seja, sem realização de triagem prévia, foi capaz de reduzir a incidência de infecções do sítio cirúrgico (5 *versus* 15 casos; 0,2 *versus* 0,8%; *p* = 0,013) e também de IAP associada a *S. aureus* . Diante dos resultados, considerou-se que o custo-benefício seria válido representando ganhos econômicos e menor morbimortalidade associada a infecções pós artroplastias. 33 Em ambos, não foram identificados parâmetros preditivos úteis para a implementação da estratégia de triagem, sendo que a descolonização universal seria efetiva e apropriada nos casos de pacientes submetidos a artroplastia de quadril. 25 29 33

Dentre os estudos selecionados, também foi abordada a antibioticoprofilaxia, discutindo aspectos relacionados ao tipo de fármaco a ser empregado, bem como variáveis levadas em consideração para início e descontinuação. Na Turquia, observou-se que a maior parte dos cirurgiões (56,8%) utilizam antibioticoprofilaxia por tempo superior ao proposto nas diretrizes (> 24 horas), a avaliação glicêmica antes da cirurgia foi realizada por 94% da amostra e a descolonização para MRSA foi realizada por 33,3% dos participantes do estudo. 34 A administração oportuna de antibióticos e ausência de comorbidades foram associados a um desfecho favorável tanto na prevenção quanto no controle de IAP. 35
